# Planning for Outcomes (P_4_O) Modeling Tool: Estimating the Impact of Changing the Proportion of Injectable Progestins in the Contraceptive Method Mix

**DOI:** 10.9745/GHSP-D-19-00062

**Published:** 2019-06-24

**Authors:** Elena Lebetkin, Xiaoming Gao, Douglas Taylor, Lauren Y. Maldonado, Abdulmumin Saad, Markus J. Steiner, Laneta J. Dorflinger, Kavita Nanda, Timothy D. Mastro

**Affiliations:** a FHI 360, Durham, NC, USA.; b United States Agency for International Development, Washington, DC, USA.

## Abstract

**Background:**

Observational studies raise concern about a potential link between injectable progestin contraceptive use and HIV acquisition risk. This possible link is particularly relevant in sub-Saharan Africa where HIV risk is high and the method mix is skewed toward injectables. We developed the Planning for Outcomes (P_4_O) model (https://planning4outcomes.ctiexchange.org/) to predict changes in maternal and child health (MCH) and HIV outcomes that could occur if the proportion of injectables in the method mix is changed.

**Methods:**

P_4_O incorporates evidence-based assumptions to predict yearly changes in unintended pregnancies, morbidity/mortality, HIV infections (women and infants), and anticipated health care costs associated with changing the proportions of injectable users in 22 selected countries. Users of this model designate all countries or a subset and adjust inputs including percentage of injectable users who discontinue, percentage of discontinuers who begin use of an alternative method, hazard ratio for HIV infection with injectable use, method mix used by injectable discontinuers, annual probabilities of method-specific pregnancy and mother-to-child transmission of HIV, condom effectiveness against HIV, risk of HIV during pregnancy, and HIV incidence among women of reproductive age.

**Results:**

Illustrative results from all sub-Saharan African countries combined and from selected countries demonstrate the potential of P_4_O to inform program planning and procurement decisions. In countries with high use of long-acting reversible contraception, the removal of injectables from the method mix is associated with improvement in MCH and HIV indicators if most injectable users switch to more effective methods (e.g., implants). In countries with high use of short-acting methods (e.g., condoms), the model predicts mostly negative MCH outcomes.

**Conclusions:**

Policy makers and program planners may use P_4_O to inform programming and policy decisions. In all scenarios, programmatic preparation to accommodate changes to the contraceptive method mix, considerations of how the individual desires of women will be addressed, and potential burden of anticipated MCH-related costs warrant advanced consideration.

## Abstract (precis)

The interactive deterministic online modeling tool P_4_O allows users to estimate how changing the proportion of injectable progestins in the contraceptive method mix might affect HIV and maternal and child health outcomes. With careful consideration for women's individual choices, policy makers and program planners may use country-specific results to help inform programming and policy decisions.

## BACKGROUND

Access to a range of contraceptive methods is essential for voluntary family planning programs worldwide. Recent analyses demonstrate that for each additional method accessible to at least half the population in a given country, contraceptive use may increase by as much as 8%.^1^ In many countries, however, the contraceptive method mix is skewed toward a few methods, with the progestin-only injectable contraceptive depot medroxyprogesterone acetate (DMPA) dominating the method mix in many sub-Saharan African countries.^2^

Observational studies raised concern for a potential link between the use of progestin-only injectable contraceptives, particularly DMPA, and the risk of HIV acquisition. A recent systematic review estimated DMPA users may have a 40% increased risk of HIV acquisition compared with nonusers of hormonal contraception.^3^ The authors considered these observational studies to be “… informative but with important limitations to acknowledge that all studies to date are vulnerable to residual or uncontrolled confounding.”^3^

In response to these concerns, the World Health Organization (WHO) recently changed the Medical Eligibility Criteria for Contraceptive Use (MEC) for progestin-only injectable use among women at high risk for HIV from a 1 (no restrictions) to a 2 (advantages generally outweigh the risks).^4^ With results expected in mid-2019, the randomized clinical trial, the Evidence for Contraceptive Options and HIV Outcomes (ECHO) Study, will provide high-quality data on the relative risks of HIV acquisition among African women randomized to use DMPA, the progestin implant Jadelle, or a copper intrauterine device (IUD). ^5^

Any potential link between DMPA use and HIV acquisition may critically affect many developing countries, particularly in sub-Saharan Africa where HIV risk is high and the contraceptive method mix is heavily skewed toward injectable progestins. Policy makers in countries with a high HIV burden may inappropriately choose to restrict injectable availability and provision if a significant association is confirmed. While restricting injectable contraceptive availability may lead to fewer HIV infections, this benefit may be offset if women who stop using injectable contraceptives have an unintended pregnancy and are at risk of negative sequelae, including maternal mortality. Moreover, if women with HIV infection who were using injectables move to less effective methods, the number of children born with HIV or acquire HIV in infancy could increase. Preparing for the impact of these theoretical changes in key maternal and child health (MCH) and HIV outcomes, as well as the programmatic desire to ensure women who discontinue injectables are provided alternative method choices to meet their needs, is integral to effective program planning and product procurement.

The literature contains several predictive models that examine the impact of a positive association between injectable hormonal contraceptive use and HIV acquisition risk on MCH and HIV outcomes in sub-Saharan Africa. Jain^6^ used data from sub-Saharan African countries on competing risks of unwanted birth and HIV acquisition associated with the use of various contraceptive methods to model ratios of additional unwanted births and additional maternal deaths per 100 HIV infections averted. Similarly, Butler et al.^7^ explored country-level effects of reducing injectable hormonal contraceptive use among women of reproductive age on the number of HIV infections, live births, and resulting net consequences on HIV/AIDS deaths and maternal mortality. Lastly, Rodriguez et al.^8^ developed a decision-analytic model to compare the benefits and risks of progestin-only injectable use on competing risks of maternal mortality and HIV acquisition on life expectancy in 9 African countries. Our model builds upon these existing tools to offer users an interactive, freely available online interface with adjustable inputs to predict a wide variety of MCH, HIV, and health cost-related outcomes (Box). This tool, Planning for Outcomes (P_4_O), is available at https://planning4outcomes.ctiexchange.org/.

BOX **What Is Unique About Planning for Outcomes?**

Planning for Outcomes (P_4_O) is an interactive, freely available online tool that allows users to adjust inputs to model key outputs pertaining to maternal and child health and HIV. It is distinct from previous modeling exercises in several notable ways:

- P_4_O incorporates method-specific pregnancy rates and country-specific method mixes.
- P_4_O includes all women of reproductive age, not just those who are married or in union.
- P_4_O's web-based interface allows users to adjust a variety of inputs, including:
  - Country or region (i.e., all countries modeled, only sub-Saharan African countries, or individual countries)
  - Assumed hazard ratio for HIV infection among injectable users relative to no contraceptive method
  - Proportion of injectable users who adopt other methods
  - How women are reallocated to the existing method mix
  - Additional inputs (i.e., HIV incidence, maternal-to-child transmission, method effectiveness, risk of HIV during pregnancy)
- Models a wide variety of outcomes, including (but not limited to):
  - Unintended pregnancies
  - Live births
  - Induced abortions
  - Unsafe abortions (subset of induced abortions)
  - Maternal deaths
  - HIV infections (among women of reproductive age)
  - Children with HIV (from maternal to child transmission)
  - Maternal and neonatal health costs

We developed P_4_O to enable model users to estimate the impact of changing the amount of injectable progestin use (as a proportion of the method mix) on key MCH and HIV outcomes. P_4_O is an interactive tool that facilitates policy and program planning decisions and enables countries to better prepare for theoretical anticipated changes. Although P_4_O will show what is expected to happen mathematically if changes to the method mix occur, programmatic decisions about method changes should be driven by a desire to ensure women who access these programs can make voluntary and informed individual choice. Estimates derived from P_4_O help highlight the programmatic challenges that may arise when a preferred method of contraception is removed from the contraceptive method mix, including, but not limited to, the potential need for additional training of providers. Addressing these challenges proactively is essential to continuing to provide family planning services that are truly guided by voluntarism and informed choice.

P_4_O serves as an interactive tool for policy makers and program planners to estimate the impact of changes in the proportion of injectable contraceptives in the method mix on HIV and MCH outcomes.

## METHODS

### Model Overview

**FIGURE 1:**
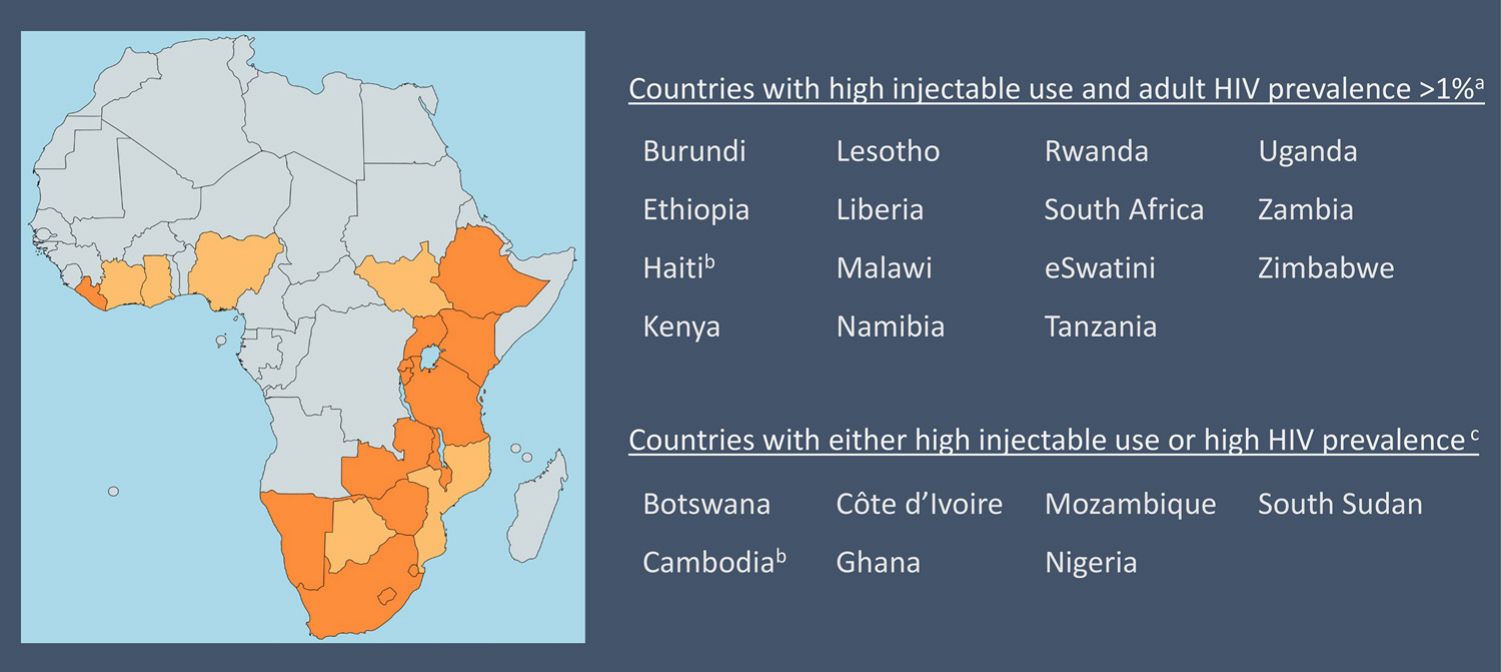
Countries Included in the Planning for Outcomes (P_4_O) Model (N=22) ^a^ Countries with high injectable use and adult HIV prevalence >1% are depicted in dark orange. ^b^ Cambodia and Haiti are included in the model but not featured in the map above. ^c^ Countries with either high injectable use or high HIV prevalence are depicted in light orange.

P_4_O is an interactive deterministic model that predicts yearly changes in key MCH and HIV indicators for all women of reproductive age (ages 15–49) based on an assumption about the hazard ratio (HR) for HIV acquisition among injectable progestin users, changes to the proportion of injectable progestins in the contraceptive method mix, and redistribution of users to remaining country-specific or a user-specified method mix. In total, 22 countries are included in this model (Figure 1). We included 15 countries with high injectable progestin use as a proportion of the modern contraceptive method mix (≥25%) and an adult HIV prevalence greater than 1%, and an additional 7 countries with either high HIV prevalence or high injectable progestin use. The user can run the model to examine results by individual country, by all countries, or by all sub-Saharan African countries. Users can also adjust the presumed HR for HIV acquisition among injectable progestin users. To date, the literature does not suggest an association with HIV acquisition among users of other hormonal methods of contraception or the nonhormonal copper IUD. As such, we only allow for modifications to the risk of HIV among injectable contraceptive users.

### Flow (Inputs/Outputs)

**FIGURE 2:**
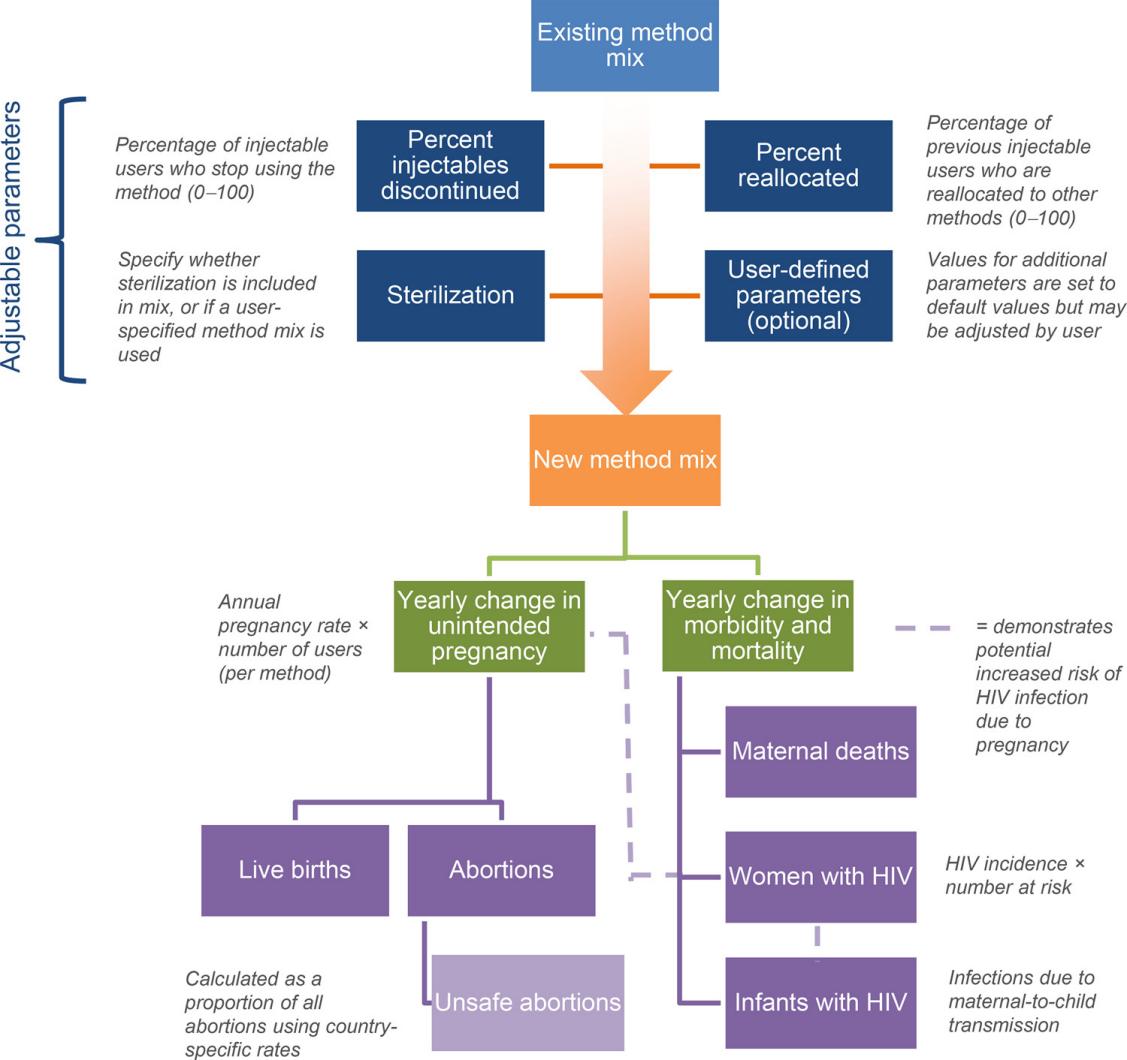
Model Flow Diagram

After designating a country context, users may modify key inputs including (Figure 2):

1. The HR for HIV acquisition with injectable progestin use (compared with no method)
2. Percentage of injectable progestin users who stop the method
3. Percentage of users reallocated to the remaining country-specific method mix (When we use the term “reallocate” to describe movement of injectable progestin users to other methods, we are referring to the mathematical reallocation of individuals to compute model results. We expect women will make individual voluntary and informed decisions on their contraception use.)
  We use the term “reallocate” to refer to the mathematical reallocation of individuals. Women make individual voluntary and informed decisions.
4. Inclusion or exclusion of sterilization

To generate country-specific method mix values, we used the most up-to-date national survey data available from a variety of sources (see Supplement). We established an HR range for HIV acquisition with injectable progestins of 0.5 to 5.0 to ensure implausible results would be avoided. We reasoned that injectable progestin users would reallocate to 1 of 3 options when they discontinue the method. In option 1, the model redistributes injectable progestin discontinuers in proportion to the existing, country-specific distribution of other modern methods, after excluding injectables. In option 2, the model redistributes discontinuers in proportion to the existing distribution of other modern methods, after excluding injectable progestins and sterilization (i.e., permanent methods such as bilateral tubal ligation and vasectomy). In option 3, the user may specify the method mix of noninjectable methods to which discontinuers are redistributed.

Key MCH and HIV-related outputs, including yearly changes in unintended pregnancy, morbidity, and mortality, are displayed in graphical or tabular form. A few outputs such as added maternal and neonatal health costs and the percentage of reallocated previous injectable users needed to balance pregnancy outcomes based on the defined method mix (“break-even point”) are summarized at the bottom of the impact panel. Yearly changes in unintended pregnancy encompasses live births, abortions, and unsafe abortions (calculated as a proportion of all abortions using regional data). Yearly changes in morbidity and mortality comprise maternal deaths, HIV infections among women of reproductive age, and children with HIV due to maternal-to-child transmission (MTCT).

### Guidance on Progestin-Only Injectable Contraception

Currently, the WHO MEC aggregates guidance for women at high risk for HIV for the 3 progestin-only injectables (intramuscular [IM] DMPA, subcutaneous [SC] DMPA, and norethindrone/norethisterone enanthate [NET-EN]).^4^ A recent review of available data on DMPA and NET-EN suggests that these 2 methods should be disaggregated in the WHO MEC guidance because they have important differences that “may plausibly result in differential impact on HIV susceptibility in women.”^9^ No data exist to support such a recommendation on the disaggregation of DMPA-IM and DMPA-SC.^9^ While we note the recommendations in this recent review, to be consistent with the current WHO MEC guidance, P_4_O does not distinguish between the different types of progestin-only injectables (henceforth “injectables”).

### Key Assumptions

P_4_O models outcomes based on a series of evidence-based assumptions (Table 1, Supplement). For our MCH indicators, we assumed all women have the annual probability of pregnancy associated with the method to which they are redistributed, and contraceptive prevalence data are consistent with the most recent national survey.^10^^–^^13^ We used estimates derived from Marie Stopes International's Impact 2 Calculator and “Adding It Up” publication to quantify impact on MCH indicators including maternal mortality, MCH health costs, and probabilities of live births and abortions.^15^^,^^19^ Furthermore, we assumed women using modern contraception or those with an unmet need due to withdrawal of injectables have at most 1 unintended pregnancy per year and stop using contraception while pregnant. In the event of unintended pregnancy, we assumed women contributed either 12 months of risk-time during pregnancy and postpartum if pregnancy resulted in a live birth, or 6 months of risk-time if the pregnancy did not result in live birth.

**TABLE 1.** Sources for Default Assumptions in the P_4_O Model

Measure	Source
Contraceptive prevalence	Demographic and Health Surveys^10^; Multiple Indicator Cluster Surveys^11^; Performance Monitoring and Accountability 2020 surveys^12^
Pregnancy rates	Contraceptive Technology^13^; Family Planning Global Handbook^14^; Adding It Up: Investing in Contraception and Maternal and Newborn Health^15^
Probability of MTCT	Kuznik et al.^16^; World Health Organization^17^
HIV prevalence and ART coverage	Joint United Nations Programme on HIV and AIDS^18^
Maternal mortality	MSI Impact 2 calculator (v.4)^19^
Maternal and neonatal health costs; live births and abortions	Adding It Up: Investing in Contraception and Maternal and Newborn Health^15^

Abbreviations: ART, antiretroviral therapy; MSI, Marie Stopes International; MTCT, mother-to-child-transmission; P_4_O, Planning for Outcomes.

P_4_O models outcomes based on a series of evidence-based assumptions.

For our HIV indicators, most default assumptions (except country-specific HIV prevalence) are modifiable by the user. We assumed HIV prevalence, antiretroviral treatment (ART), and MTCT data are consistent with point estimates provided by AIDSInfo.^16^^–^^18^ For each country, we set the pooled HIV incidence among women using contraception to a fixed fraction (default value: 10%) of the HIV prevalence among women of reproductive age. Due to lack of HIV incidence data among women of reproductive age who use modern contraception, we chose to default to the assumption used by Butler et al.^7^ that incidence is 10% of prevalence in a stable epidemic. We assumed condom users have additional protection against HIV (default value: 85% effective).^6^ Lastly, we assumed no differential risk of HIV during pregnancy, except due to discontinuation of condoms or injectable use. Some existing data suggest an increased risk of HIV acquisition during pregnancy; thus, this value is modifiable.^20^

### Uncertainty of Estimates

The model estimates are based on a large array of input parameters, all of which are associated with varying degrees of uncertainty. We recommend that the user explore this uncertainty by varying the model assumptions such as the HR for HIV associated with injectable use, the proportion of injectable users who would stop using the method, and the proportion who would adopt a new method. The assumption about HIV incidence among women of reproductive age using modern contraception and MTCT probabilities are also important drivers of model results, and these can be modified by the user, as well.

### Underlying Model Structure

In order to concisely explain the model, here we describe *only* the underlying model structure for determining MCH and HIV-related outcomes when injectable discontinuers are reallocated to methods in proportion to the existing, country-specific distribution of other modern methods, after excluding injectables. As indicated earlier, however, the model allows an option of defining the method mix alternatively.

#### Determining Estimates for Contraceptive and MCH Measures

The model assumes there are N_WRA_ women of reproductive age, and that P_INJ_ is the proportion using injectable contraception. We wish to estimate the change in the number of pregnancies per year if a proportion Y of the N_WRA_·P_INJ_ injectable users stopped using the method, when we further assume a proportion X of those who stop take up a replacement modern method. Among previous injectable users shifting to a new method, the proportion switching into each type is:

$$\begin{array}{l} Q_{i}=P_{i}/\left(P_{FS}+P_{MS}+P_{OC}+P_{IUD}+P_{MC}+P_{VB}+P_{IMP}+P_{OTH}\right),\\ i\in \left\{FS,MS,OC,IUD,MC,VB,IMP,OTH\right\}, \end{array}$$

where the terms in the denominator denote the current proportion (P) using female sterilization (FS), male sterilization (MS), oral contraceptive pills (OC), IUD, male condoms (MC), vaginal barriers (VB), implants (IMP), or other modern methods (OTH).

Next, the model allows PP_j_, where j is {*NM*, *FS*, *MS*, *INJ*, *OC*, *IUD*, *MC*, *VB*, *IMP*, *OTH*}, denoting the yearly probability of pregnancy when using no method (NM) and so forth. Based on this assumption, the change in the expected number of pregnancies per year, if a proportion Y of injectable users stop using the method and a proportion X of those who stop adopt a new method, is given by:

$$\begin{array}{c} NP_{diff}=N_{WRA}P_{INJ}Y\left(1-X\right)PP_{NM}\\ +N_{WRA}P_{INJ}YX\left(PP_{FS}Q_{FS}+PP_{MS}Q_{MS}\right)\\ +PP_{OC}Q_{OC}+PP_{IUD}Q_{IUD}+PP_{MC}Q_{MC}\\ \begin{array}{l} +PP_{VB}Q_{VB}+PP_{IMP}Q_{IMP}+PP_{OTH}Q_{OTH})\\ -N_{WRA}P_{INJ}YPP_{INJ}, \end{array} \end{array}$$

where the first line is the expected number of pregnancies among women switching to no method, the subsequent added variables (from the second line through the fourth line) amount to the expected number of pregnancies among women switching to the existing method mix, and the last line is the expected number of pregnancies that would have occurred among injectable users had they not stopped using the method. All other pregnancy-related indicators are obtained by multiplying NP_diff_ by the appropriate factor (i.e., the chance an unintended pregnancy leads to a live birth, abortion, unsafe abortion, maternal death, or additional maternal and neonatal health care costs/year).

#### Determining Estimates for HIV-Related Measures

To determine outcomes for HIV-related measures in scenarios in which the HR for HIV acquisition with injectable use is greater or less than 1.0, the user must first input an assumption about the overall incidence of HIV among nonpregnant women using modern contraception (denoted I_HIV_). Once I_HIV_ is specified, P_4_O calculates distinct HIV incidence values for condom users, injectable users, and users of methods besides condoms or injectables. The model denotes the HR for injectable use versus any method besides condoms as HR_INJ_, and the HR for condoms versus any method besides injectables as HR_MC_. Then, the incidence of HIV among users of any method besides condoms or injectables is approximated as:

$$I_{HIV}^{0}=\frac{I_{HIV}}{\left\{\left(1-Q_{INJ}-Q_{MC}\right)+HR_{MC}Q_{MC}+HR_{INJ}Q_{INJ}\right\}},$$

where Q_INJ_ is the proportion of the existing method mix, which is injectables, and Q_MC_ is the proportion of the method mix, which is male condoms. Then the incidence of HIV among injectable users is:

$$I_{HIV}^{INJ}=I_{HIV}^{0}\cdot HR_{INJ}$$

and the incidence of HIV among condom users is:

$$I_{HIV}^{MC}=I_{HIV}^{0}\cdot HR_{MC}.$$

To compute the yearly change in the number of women becoming infected with HIV, if a proportion Y of current injectable users are withdrawn from the method and a proportion X of those who stop adopt a new method, we also need to know the prevalence of HIV (PREV_HIV_) (since only those not already infected can become newly infected); the HR for HIV when a woman is pregnant or in the first few months postpartum (HR_PREG_) (since we want to allow for the possibility that HIV acquisition risk changes during this period); and the chance that an unintended pregnancy results in a live birth (F_T_) (since how long a pregnant woman is at differential risk of HIV will depend on whether she carries to term and has a live birth). P_4_O makes the simplifying assumption that women who become pregnant and carry the pregnancy to term contribute up to 1 year of HIV risk while pregnant; women who become pregnant but do not carry to term contribute 6 months of risk while pregnant and 6 months while not pregnant; women stop using their method (including condoms) while pregnant; and women who do not become pregnant contribute 1 year of HIV risk using their contraceptive method. P_4_O then computes the expected change in the yearly number of women becoming infected with HIV as:


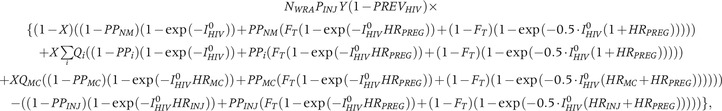

where the sum in the third row indexes women adopting {*FS, MS, OC, IUD, VB, IML, OTH*} (with or without becoming pregnant), and the last row captures the number of new infections that would have occurred among the *N_WRA_P_INJ_Y*(1-*PREV_HIV_*) women had they not stopped using injectables.

To estimate the number of additional children born with HIV if injectable use is reduced, the model determines what percentage of the extra live births were to women with HIV, what percentage of women acquire HIV while pregnant, and the probability infection is transmitted to the child. For the latter, the model must consider the percentage of women with HIV who are on ART (*PREV*_ART_), the risk of transmitting HIV to a child when on daily ART (*P_T_^ART^*), and the risk of transmitting HIV to the child when not on ART (*P_T_^0^*). Then, the excess number of children born with HIV is given by:

$$NP_{diff}\cdot P_{LB}\cdot \left\{PREV_{HIV}\left[PREV_{ART}\cdot P_{T}^{ART}+\left(1-PREV_{ART}\right)\cdot P_{T}^{0}\right]+\left(1+PREV_{HIV}\right)\left(1-\exp\left(-I_{HIV}^{0}HR_{PREG}\right)\right)\left[PREV_{ART}\cdot P_{T}^{ART}+\left(1-PREV_{ART}\right)\cdot P_{T}^{0}\right]\right\}.$$

## RESULTS

### Illustrative Scenarios

Since P_4_O is interactive and allows the user to adjust multiple inputs simultaneously, we have selected 4 illustrative examples to demonstrate how the model operates and its potential to inform programmatic decisions. We selected 3 countries with distinct contraceptive method mixes and HIV scenarios—Ethiopia, South Africa, and Zimbabwe—to demonstrate the impact of changing the proportion of injectables in settings with varied short-acting and long-acting method use. Additionally, we modeled aggregated outcomes for all 20 of the included countries in sub-Saharan Africa because we predicted that a positive association between injectable use and HIV acquisition would critically impact MCH and HIV indicators in these countries.

In all 4 scenarios, we assumed an HR for HIV with injectable use of 1.4, a 75% discontinuation of injectables, and 25% reallocation proportional to the current country-specific modern method mix after excluding permanent methods (sterilization). All other modifiable inputs remained at the default values. We chose an HR of 1.4 based on the current literature and included the other parameters to model scenarios with demonstrable impact on MCH and HIV indicators.^3^ We assumed that if restrictions were placed on injectable contraceptive use, 75% of users would stop the method, and we assumed fewer than 50% would select a new method due to likely real-life programmatic challenges in responding to rapid increases in the resulting method demand. A change in the contraceptive method mix due to women choosing to move from short- to long-acting reversible contraceptives (LARCs) may present commodity and provider-related challenges (i.e., skill set and availability of providers to provide method). Lastly, we excluded permanent methods because bilateral tubal ligation and vasectomy procedures account for a minority of the method mix in most countries modeled. Model outputs are summarized for each example below and in Table 2.

**TABLE 2.** Key MCH and HIV outcomes for All sub-Saharan African Countries and for Ethiopia, South Africa, and Zimbabwe^a^

Indicator	Ethiopia	South Africa	Zimbabwe	All sub-Saharan Africa^b^
**Yearly change in unintended pregnancies, No.**				
Pregnancies	824,933	744,963	51,341	3,565,329
Live births	464,583	374,107	28,914	1,911,731
Abortions	243,121	269,123	15,131	1,155,684
Unsafe abortions	184,976	71,163	11,512	749,343
**Yearly change in morbidity and mortality, No.**				
Women with HIV	−1,227	−22,866	−994	−44,450
Infants with HIV	988	5,560	329	16,068
Maternal deaths	2,750	883	167	12,062
**Reallocated DMPA users per method, No.**				
Pill	149,344	152,698	30,606	742,767
IUD	43,559	27,486	453	179,190
Male condom	26,965	357,313	4,761	823,462
Implant	495,739	97,727	9,182	1,256,796
Other^c^	37,336	6,108	340	160,046
**Additional maternal and neonatal health care costs per year, US$**	35,112,706	118,779,370	2,185,284	249,429,105

Abbreviations: DMPA, depot medroxyprogesterone acetate; HR, hazard ratio; IUD, intrauterine device; MCH, maternal child health. Note: We are updating the P_4_O model as new data become available. Thus, results produced may be different from results displayed in this table.

a Assumptions: HR for HIV with DMPA=1.4; 75% of injectable users discontinue; 25% reallocate to other methods according to country-specific method mix after excluding permanent methods (sterilization); other parameters set to default.

b Included sub-Saharan African countries: Botswana, Côte d'Ivoire, Ghana, Mozambique, Nigeria, South Sudan, Burundi, Ethiopia, Kenya, Lesotho, Liberia, Malawi, Namibia, Rwanda, South Africa, eSwatini, Tanzania, Uganda, Zambia, and Zimbabwe.

c Other methods include emergency contraception, Lactational Amenorrhea Method, Standard Days Method, and other modern methods.

#### Ethiopia

Ethiopia exemplifies a country setting with relatively low HIV prevalence (1.3% among women of reproductive age) and high use of LARCs (i.e., implants and IUDs). Currently, injectables make up 63.0% of the method mix; LARCs make up 26.1%; and short-acting methods other than injectables (i.e., contraceptive pills or male condoms) make up 10.3% of the method mix. In our modeled scenario, nearly 500,000 previous injectable users switch to implants and nearly 150,000 switch to contraceptive pills. In this scenario, over 75% of discontinuers shift to a more effective LARC within the existing method mix. In the setting of low HIV prevalence, the impact of these changes in the method mix on HIV acquisition among women is lower than in countries with a high HIV prevalence, with approximately 1,200 fewer women acquiring HIV. Additionally, the model predicts a yearly increase of approximately 243,000 abortions, nearly 76% of which are anticipated to be unsafe.

In the setting of low HIV prevalence, the impact of injectable discontinuers shifting to a more effective LARC on HIV acquisition is lower than in countries with high HIV prevalence.

#### South Africa

In contrast to Ethiopia, South Africa is notable for its high HIV prevalence—23.8% among women of reproductive age—and mixed use of long- and short-acting contraceptive methods. Injectable contraceptives account for 47.3% of the modern contraceptive method mix, followed by male condoms (24.5%), contraceptive pills (10.5%), and sterilization (8.8%). Among method users, 8.6% use a LARC for contraception. In this modeled scenario, most injectable discontinuers shift to short-acting methods; the model predicts approximately 357,000 and 153,000 discontinuers will switch to male condoms and contraceptive pills, respectively. The model predicts nearly 750,000 unintended pregnancies, over 370,000 live births, and over 269,000 abortions. Further, in the setting of high HIV prevalence, the changes to the method mix are expected to result in approximately 23,000 fewer cases of HIV acquisition among women. Of note, the additional maternal and neonatal health care costs per year are substantially higher than the other 2 countries modeled. Cost estimates, derived from both direct costs, such as personnel time, commodities, medical care, and counseling, and indirect costs, such as program management, health education, advocacy, and infrastructure improvements, are significantly higher in the southern African region.^15^

#### Zimbabwe

The prevalence of contraceptive pill use in Zimbabwe creates a unique scenario for modeling outcomes. Contraceptive pills currently account for 56.5% of the modern contraceptive mix, followed by implants (16.9%), injectables (15.1%), and male condoms (8.8%). Among contraceptive users, 17.7% rely on LARCs for contraception. The HIV prevalence among women of reproductive age is relatively high, 16.1%, although substantially lower than that of South Africa. Current injectable use is also the lowest among the 3 countries modeled. In this scenario, most women discontinuing injectable use switch to using a less effective contraceptive method (pills)—more than 3 times as many as those predicted to switch to implants. As women move to less effective methods, unintended pregnancy is expected to increase significantly, with over 51,000 additional unintended pregnancies expected. However, in Zimbabwe, where HIV prevalence is high but injectable use is modest, adjusting the contraceptive use in this scenario results in nearly 1,000 fewer female cases of HIV acquisition.

#### All sub-Saharan African Countries

In sub-Saharan Africa, there are an estimated 153,113,000 women of reproductive age, with an overall HIV prevalence of 7.1%. Injectables dominate the method mix in aggregated sub-Saharan African countries, with 41.8% of contraceptive users using injectables, followed by implants (16.4%), male condoms (16.1%), pills (14.5%), sterilization (5.1%), and IUDs (2.8%). In this scenario, using 20 countries in the region, the model predicts most reallocated injectable discontinuers will switch to implants (approximately 1,257,000 women) or to male condoms (approximately 823,000 women). In this scenario, approximately 44,000 fewer HIV infections among women are expected; however, this impact is underscored by a predicted 3,565,000 additional pregnancies and nearly 2,000,000 abortions. These outcomes in turn affect the overall added maternal and child health care costs, predicted to total at approximately US$249,429,000.

## DISCUSSION

This P_4_O model serves as a planning tool for policy makers and program planners to input realistic country-specific scenarios and use results to guide contraceptive programming and policy-related decisions. Additionally, the online interface of the model, along with the addition of a variety of instructional materials, makes P_4_O approachable, accessible, and easily adjustable by a diverse range of users. In most plausible injectable redistribution scenarios, any predicted population-level benefits of reduced HIV incidence that occur by removing injectables from the contraceptive method mix would need to be balanced against the negative public health impacts expected for other outcomes, including increases in unintended pregnancies, abortions, maternal deaths, and HIV infections in children. These findings reflect those in other published modeling work on MCH and HIV outcomes related to injectable contraceptive discontinuation.^6^^–^^8^ Rodriquez et al.^8^ stated:

*… removal of (progestin-only injectable) contraception from the market without effective and acceptable contraception replacement would have a net negative effect on maternal health, life expectancy, and mortality under a variety of scenarios.*

However, the outcome of redistribution scenarios is highly dependent on the contraceptive method mix, with more encouraging outcomes expected when women have access to LARCs in the method mix. Increasing the availability of and access to LARC methods may mitigate the public health impact of restricting injectables if DMPA is found to be significantly associated with HIV acquisition. However, changing the contraceptive method mix while ensuring informed and voluntary choice is not a simple task and takes advance preparation because providing increased access to LARCs has significant programmatic, financial, and logistical challenges.

Increasing the availability of and access to LARCs may mitigate the public health impact of restricting injectables if DMPA is found to be significantly associated with HIV acquisition.

In the scenarios modeled, one may highlight the relative impact of reallocation on potential challenges in product procurement. In each country setting, the demand for rapid procurement of methods to account for the number of previous injectable users switching to other methods is substantial and worthy of advanced consideration. In Ethiopia, for instance, the model predicts nearly half a million previous injectable users will shift to implants. Aggregated results from all 20 sub-Saharan African countries reveal similar trends, with more than 1,257,000 previous injectable users predicted to switch to implants. Family planning programs may use this information to determine whether key factors such as local demand and knowledge, supply, service provision, access to removal services, and other implicated costs are adequately addressed on the timeline needed to prepare for this transition.

Apart from the model's implications in the setting of HIV acquisition risk, P_4_O may also serve as a tool for understanding the impact of method skew. In many countries, 50% or more contraceptive users rely on a single method for contraception. By this convention, both Ethiopia and Zimbabwe demonstrate contraceptive use patterns consistent with method skew. South Africa comes close with nearly 50% of the method mix attributable to injectable contraceptives. When most users rely on a single method, it may reflect supply-chain-related challenges in which programs only offer 1 or 2 contraceptive methods rather than the full range of those available. Method skew is attributable to many factors, including but not limited to client characteristics (i.e., age or life stage, desire for limiting versus spacing births), method characteristics (i.e., cost, ease of use, popularity), history (i.e., length of time since introduction of method), provider bias, and policies and programs more broadly.^2^^,^^21^ Although positive method characteristics may influence skew, heavily relying on a few contraceptive methods may cause a myriad of downstream challenges if there are sudden, mass shifts between methods. These trends are present in our modeled scenario, which reveal most users in each country setting will disproportionately move from injectables to 1 or 2 methods, namely a combination of contraceptive pills, implants, or male condoms. Countries need to closely examine their family planning programs to ensure they are prepared to cope with these potential shifts while upholding and advancing volunteerism as well as broad and informed method choice for all clients.^2^

P_4_O may also serve as a tool for understanding the impact of method skew.

### Strengths and Limitations of the P_4_O Model

To our knowledge, P_4_O is the first tool of its kind to interactively model the impact of changing the proportion of injectable contraceptive users in the contraceptive method mix. This issue is highly pertinent to the current family planning landscape as we await the ECHO study results.^5^ Our model uses evidence-based assumptions and rigorous methodology to model outcomes based on best available estimates for maternal and HIV-related indicators. Further, although the model is currently limited to outcomes specifically related to injectable discontinuation and reallocation, it can be modified to include discontinuation and reallocation of other contraceptive methods and updated data. As hazard ratios for HIV acquisition with other contraceptive methods are published in the literature, we plan to incorporate any risks associated with these methods in future iterations. Lastly, the online and interactive nature of this model facilitates greater accessibility and utility among broad audiences.

P_4_O is intended to help policy makers, family planning and HIV program planners, and other stakeholders understand the potential impact of a change in current injectable contraceptive prevalence on pregnancy and HIV outcomes. As with any model, however, flawed or implausible assumptions can lead to flawed or implausible outputs. P_4_O possesses several additional key limitations. First, the model is limited to 22 countries; we plan to expand the tool to additional countries in future modeling work. Second, P_4_O does not consider long-term changes to population size, further shifts in the method mix, or new interventions to prevent or treat HIV. Third, the model does not consider the impact of pre-exposure prophylaxis or condom use, which may differ by contraceptive method. Lastly, the model does not distinguish between injectable progestins, which may have different risks.^9^ We plan to update P_4_O if and/or when data differentiating risks among injectable progestins are available.

## CONCLUSIONS

As the world awaits results clarifying data from a randomized controlled trial on the relationship between DMPA use and HIV acquisition, programs must begin to consider downstream implications of any negative findings. We hope this model attracts and is useful to a variety of users with diverse interests—health care policy makers, ministry of health officials, family planning and HIV program planners, commodities procurers, advocacy groups, funders, and those with general interest in global family planning. Regardless of how different audiences choose to use this tool, we underscore the importance of individual choices when planning for potential outcomes and urge programs to consider these results and their implications when making programmatic and policy-related decisions.

## Supplementary Material

19-00062-Lebetkin-Supplement.pdf

## Notes

**Peer Reviewed**

**First Published Online:** June 12, 2019

**Funding:** This work is made possible by the generous support of the American people through the United States Agency for International Development (USAID), provided to FHI 360 through Cooperative Agreement AID-OAA-A-15-00045.

## References

[1] Ross J Stover J. Use of modern contraception increases when more methods become available: analysis of evidence from 1982–2009. Glob Health Sci Pract. 2013;1(2):203–212. 10.9745/GHSP-D-13-00010. 25276533 PMC4168565

[2] Bertrand JT Sullivan TM Knowles EA Zeeshan MF Shelton JD. Contraceptive method skew and shifts in method mix in low- and middle-income countries. Int Perspect Sex Reprod Health. 2014;40(3):144–153. 10.1363/4014414. 25271650

[3] Polis CB Curtis KM Hannaford PC. An updated systematic review of epidemiological evidence on hormonal contraceptive methods and HIV acquisition in women. AIDS. 2016;30(17):2665–2683. 10.1097/QAD.0000000000001228. 27500670 PMC5106090

[4] World Health Organization (WHO). Hormonal Contraceptive Eligibility for Women at High Risk of HIV. Geneva, Switzerland: WHO; 2017. https://apps.who.int/iris/bitstream/handle/10665/254662/WHO-RHR-17.04-eng.pdf. Accessed May 21, 2019.

[5] Hofmeyr GJ Morrison CS Baeten JM.; ECHO Trial Team. Rationale and design of a multi-center, open-label, randomised clinical trial comparing HIV incidence and contraceptive benefits in women using three commonly-used contraceptive methods (the ECHO study). Gates Open Research. 2017;1:17. 10.12688/gatesopenres.12775.1. 29355224 PMC5771152

[6] Jain AK. Hormonal contraception and HIV acquisition risk: implications for individual users and public policies. Contraception. 2012;86(6):645–652. 10.1016/j.contraception.2012.03.008. 22541635

[7] Butler AR Smith JA Polis CB Gregson S Stanton D Hallett TB. Modelling the global competing risks of a potential interaction between injectable hormonal contraception and HIV risk. AIDS. 2013;27(1):105–113. 10.1097/QAD.0b013e32835a5a52. 23014519 PMC4862571

[8] Rodriguez MI Gaffield ME Han L Caughey AB. Re-evaluating the possible increased risk of HIV acquisition with progestin-only injectables versus maternal mortality and life expectancy in Africa: a decision analysis. Glob Health Sci Pract. 2017;5(4):581–591. 10.9745/GHSP-D-17-00243. 29284696 PMC5752605

[9] Heffron R Achilles SL Dorflinger LJ. Pharmacokinetic, biologic, and epidemiologic differences in MPA- and NET-based progestin-only injectable contraceptives relative to the potential impact on HIV acquisition in women. Contraception. 2019;99(4):199–204. 10.1016/j.contraception.2018.12.001. 30576636 PMC6467541

[10] The DHS Program, Demographic and Health Surveys. The DHS Program website. https://www.dhsprogram.com. Accessed May 21, 2019.

[11] United Nations Children's Fund (UNICEF). Multiple Indicator Cluster Surveys (MICS). UNICEF website. https://www.unicef.org/statistics/index_24302.html. Accessed May 21, 2019.

[12] Performance Monitoring and Accountability 2020 (PMA2020). PMA2020 website. https://www.pma2020.org. Accessed May 21, 2019.

[13] Hatcher RA Nelson A Trussell J. Contraceptive Technology. 21st ed. New York, NY: Ayer Company Publishers, Inc.; 2018.

[14] World Health Organization Department of Reproductive Health and Research (WHO/RHR) and Johns Hopkins Bloomberg School of Public Health/Center for Communication Programs (CCP), Knowledge for Health Project. Family Planning: A Global Handbook for Providers. 2018 edition. Baltimore, MD, and Geneva, Switzerland: CCP and WHO; 2018. https://www.fphandbook.org/. Accessed May 21, 2019.

[15] Darroch JE. Adding It Up: Investing in Contraception and Maternal and Newborn Health, 2017—Estimation Methodology & Methodology Tables. New York, NY: Guttmacher Institute; 2018. https://www.guttmacher.org/report/adding-it-up-investing-in-contraception-maternal-newborn-health-2017-methodology. Accessed May 21, 2019.

[16] Kuznik A Lamorde M Hermans S. Evaluating the cost-effectiveness of combination antiretroviral therapy for the prevention of mother-to-child transmission of HIV in Uganda. Bull World Health Organ. 2012;90(8):595–603. 10.2471/BLT.11.095430. 22893743 PMC3417786

[17] World Health Organization (WHO). Mother-to-child transmission of HIV. WHO website. http://www.who.int/hiv/topics/mtct/about/en/. Accessed May 21, 2019.

[18] Joint United Nations Programme on HIV and AIDS (UNAIDS). Regions and countries. UNAIDS website. http://www.unaids.org/en/regionscountries. Accessed May 21, 2019.

[19] Marie Stopes International. Impact 2 Calculator. Marie Stopes website. https://mariestopes.org/impact-2. Updated March 2019. Accessed May 21, 2019.

[20] Thompson KA Hughes J Baeten JM. Increased risk of HIV acquisition among women throughout pregnancy and during the postpartum period: a prospective per-coital-act analysis among women with HIV-infected partners. J Infect Dis. 2018;218:16–25. 10.1093/infdis/jiy113. 29514254 PMC5989601

[21] Bertrand JT Rice J Sullivan TM Shelton J. Skewed Method Mix: A Measure of Quality in Family Planning Programs. Chapel Hill, NC: MEASURE Evaluation; 2000. https://www.measureevaluation.org/resources/publications/wp-00-23. Accessed May 21, 2019.

