# Supplementary material for: Planning for Outcomes (P4O) Modeling Tool: Estimating the Impact of Changing the Proportion of Injectable Progestins in the Contraceptive Method Mix
Source: Glob Health Sci Pract. 2019 Jun 24;7(2):317–28. doi: 10.9745/GHSP-D-19-00062 (PMC6641814; doi:10.9745/GHSP-D-19-00062)
Supplement: 19-00062-Lebetkin-Supplement.pdf [file 19-00062-Lebetkin-Supplement.pdf]

P4O
planning for outcomes

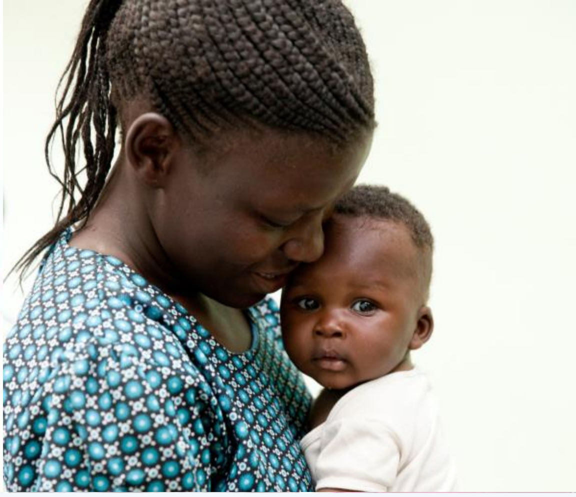

Planning for Outcomes (P_4O) Model

DATA SOURCE REFERENCES

2018 version

Unintended Pregnancies

- Calculated from yearly probability of pregnancy as per contraceptive method mix.
- Yearly probability of pregnancy per method:
  - Hatcher RA, Nelson AL, Trussell J, Cwiak C, Carson P, Policar MS, Edelman A, Aiken ARA, Marrazzo J, Kowal D, eds. *Contraceptive technology.* 21st edition. New York, NY: Ayer Company Publishers, Inc. 2018.
  - World Health Organization Department of Reproductive Health and Research (WHO/RHR) and Johns Hopkins Bloomberg School of Public Health/Center for Communication Programs (CCP), Knowledge for Health Project. Family Planning: A Global Handbook for Providers (2018 update). Baltimore and Geneva: CCP and WHO, 2018. Available from: https://www.fphandbook.org/
- Probability of pregnancy among women with unmet need for contraception:
  - Darroch JE, *Adding It Up: Investing in Contraception and Maternal and Newborn Health, 2017—Estimation Methodology & Methodology Tables*, New York: Guttmacher Institute, 2018. Available from: https://www.guttmacher.org/report/adding-it-up-investing-in-contraception-maternal-newborn-health-2017-methodology

Maternal Deaths

- Impact 2 (v4), *Marie Stopes International,* 2016
- Source data: Maternal deaths
  - WHO. Trends in Maternal Mortality: 1990 to 2008 Estimates from WHO, UNICEF, UNFPA, and The World Bank Geneva: World Health Organisation, 2010.

MTCT Probabilities

- Kuznik A, Lamorde M, Hermans S, Castelnuovo B, Auerbach B, Semeere A, et al. Evaluating the cost-effectiveness of combination antiretroviral therapy for the prevention of mother-to-child transmission of HIV in Uganda. Bulletin of the World Health Organization. 2012;90(8):595-603. Available from: http://www.who.int/bulletin/volumes/90/8/11-095430/en/
- World Health Organization. Mother-to-child transmission of HIV [Internet]. 2018 [cited 10 Jan 2018]. Available from: http://www.who.int/hiv/topics/mtct/about/en/

USAID
Envision FP
fhi360 THE SCIENCE OF IMPROVING LIVES

Live births & Abortions

- Darroch JE, *Adding It Up: Investing in Contraception and Maternal and Newborn Health, 2017—Estimation Methodology & Methodology Tables*, New York: Guttmacher Institute, 2018. Available from: https://www.guttmacher.org/report/adding-it-up-investing-in-contraception-maternal-newborn-health-2017-methodology
- Source data:
  - Live births
    - Bearak J et al., Global, regional, and subregional trends in unintended pregnancy and its outcomes from 1990 to 2014: estimates from a Bayesian hierarchical model, Lancet Global Health, 2018, 6(4):e380–e389, doi:10.1016/S2214-109X(18)30029-9.
  - Abortions
    - *Induced:* Sedgh G et al., Abortion incidence between 1990 and 2014: global, regional, and subregional levels and trends, Lancet, 2016, 388(10041):258–267, doi:10.1016/S0140-6736(16)30380-4.
    - *Unsafe:* Ganatra B et al., Global, regional, and subregional classification of abortions by safety, 2010–14: estimates from a Bayesian hierarchical model, Lancet, 2017, 390(10110):2372–2381, doi:10.1016/S0140-6736(17)31794-4.

HIV data

- *Prevalence, ART coverage, MTCT coverage*
  - UNAIDS. AIDSInfo [Internet]. 2018 [cited 10 Jan 2018]. Available from: http://aidsinfo.unaids.org/
- *Incidence* (divide prevalence by 10 – same method used in Butler AR, Smith JA, Polis CB, Gregson S, Stanton D, Hallett TB. Modelling the global competing risks of a potential interaction between injectable hormonal contraception and HIV risk. AIDS. 2013 Jan 2;27(1):105-113.)

Population (women of reproductive age 15-49)

- United States Census Bureau. International Data Base [Internet]. August 2017 [cited 10 Jan 2018]. Available from: https://www.census.gov/data-tools/demo/idb/informationGateway.php

Cost for modern contraceptive and maternal and newborn health care

- Darroch JE et al., Adding It Up: Investing in Contraception and Maternal and Newborn Health, 2017—Supplementary Tables, New York: Guttmacher Institute, 2017, www.guttmacher.org/fact-sheet/adding-it-up-contraception-mnh-2017 (numbers in model were derived by dividing the MNH service costs for unintended pregnancies from Table 8 (Columns E and J) the total number of unintended pregnancies from Table 3)

Contraceptive prevalence rate

**Note: South Africa is only country with 2-month injectable use reported. For purposes of model, 2- and 3-month injectables are combined into 3-month injectables*

- Botswana - Central Statistics Office (Botswana). Botswana Family Health Survey 2007-2008. Gaborone, Botswana: Central Statistics Office (Botswana), 2009. Available from: http://www.asclibrary.nl/docs/338151265.pdf
- Burundi- Institut de Statistiques et d'Études Économiques du Burundi - ISTEEBU, Ministère de la Santé Publique et de la Lutte contre le Sida -MSPLS - Burundi, ICF International. Burundi Enquête Démographique et de Santé 2016/2017. Bujumbura, Burundi: ISTEEBU, MSPLS, and ICF International, 2016/2017.
- Cambodia - National Institute of Statistics/Cambodia, Directorate General for Health/Cambodia, and ICF International. 2015. Cambodia Demographic and Health Survey 2014. Phnom Penh, Cambodia: National Institute of Statistics/Cambodia, Directorate General for Health/Cambodia, and ICF International.
- Cote D'Ivoire - Institut National de la Statistique - INS/Côte d'Ivoire and ICF International. 2012. Enquête Démographique et de Santé et à Indicateurs Multiples de Côte d'Ivoire 2011-2012. Calverton, Maryland, USA: INS/Côte d'Ivoire and ICF International.
- Ethiopia – PMA2020. PMA2016 Ethiopia Family Planning Brief. Available from: https://www.pma2020.org/reports/pma2016ethiopia-r4-fp-brief
- Ghana – PMA2020. PMA 2016 Ghana Family Planning Brief. Available from: https://www.pma2020.org/reports/pma2016ghana-r5
- Haiti - Institut Haïtien de l'Enfance (IHE) [Haïti] et ICF. 2017. Enquête Mortalité, Morbidité et Utilisation des Services, Haïti, 2016-2017 : Indicateurs Clés. Rockville, Maryland, et Pétion-Ville, Haïti: IHE et ICF.
- Kenya – PMA2020. PMA2016 Kenya Family Planning Brief. Available from: https://pma2020.org/sites/default/files/KER5-2PG-FP-Indicator-Brief-v13-2017-07.14.sed_.pdf
- Lesotho - Ministry of Health/Lesotho and ICF International. 2016. Lesotho Demographic and Health Survey 2014. Maseru, Lesotho: Ministry of Health/Lesotho and ICF International.
- Liberia - Liberia Institute of Statistics and Geo-Information Services - LISGIS, Ministry of Health and Social Welfare/Liberia, National AIDS Control Program/Liberia, and ICF International. 2014. Liberia Demographic and Health Survey 2013. Monrovia, Liberia: LISGIS and ICF International.
- Malawi - National Statistical Office - NSO/Malawi and ICF. 2017. Malawi Demographic and Health Survey 2015-16. Zomba, Malawi: NSO and ICF.
- Mozambique - Ministerio da Saude - MISAU/Moçambique, Instituto Nacional de Estatística - INE/Moçambique and ICF International. Moçambique Inquérito Demográfico e de Saúde 2011. Calverton, Maryland, USA: MISA/Moçambique, INE/Moçambique and ICF International.

Contraceptive prevalence rate, continued.

- Namibia - The Nambia Ministry of Health and Social Services - MoHSS - and ICF International. 2014. The Namibia Demographic and Health Survey 2013. Windhoek, Namibia: MoHSS/Namibia and ICF International.
- Nigeria – PMA2020. PMA 2016 Nigeria Family Planning Brief. Available from: https://www.pma2020.org/reports/pma2016nigeria
- Rwanda- National Institute of Statistics of Rwanda, Ministry of Finance and Economic Planning/Rwanda, Ministry of Health/Rwanda, and ICF International. 2015. Rwanda Demographic and Health Survey 2014-15. Kigali, Rwanda: National Institute of Statistics of Rwanda, Ministry of Finance and Economic Planning/Rwanda, Ministry of Health/Rwanda, and ICF International.
- South Africa - National Department of Health (NDoH), Statistics South Africa (Stats SA), South African Medical Research Council (SAMRC), and ICF. 2019. South Africa Demographic and Health Survey 2016. Pretoria, South Africa, and Rockville, Maryland, USA: NDoH, Stats SA, SAMRC, and ICF.
- South Sudan - Ministry of Health and National Bureau of Statistics, 2010. South Sudan Household Survey 2010, Final Report. Juba, South Sudan. Available from: http://microdata.worldbank.org/index.php/catalog/2588
- Swaziland - Central Statistical Office/Swaziland and Macro International. 2008. Swaziland Demographic and Health Survey 2006-07. Mbabane, Swaziland: Central Statistical Office/Swaziland and Macro International.
- Tanzania - Ministry of Health, Community Development, Gender, Elderly and Children - MoHCDGEC/Tanzania Mainland, Ministry of Health - MoH/Zanzibar, National Bureau of Statistics - NBS/Tanzania, Office of Chief Government Statistician - OCGS/Zanzibar, and ICF. 2016. Tanzania Demographic and Health Survey and Malaria Indicator Survey (TDHS-MIS) 2015-16. Dar es Salaam/Tanzania: MoHCDGEC, MoH, NBS, OCGS, and ICF.
- Uganda – PMA2020. PMA 2016 Uganda Family Planning Brief. Available from: https://www.pma2020.org/reports/pma2016uganda-r4
- Zambia - Central Statistical Office (CSO) [Zambia], Ministry of Health (MOH) [Zambia], and ICF International. 2014. Zambia Demographic and Health Survey 2013-14. Rockville, Maryland, USA: Central Statistical Office, Ministry of Health, and ICF International
- Zimbabwe - Zimbabwe National Statistics Agency and ICF International. 2016. Zimbabwe Demographic and Health Survey 2015: Final Report. Rockville, Maryland, USA: Zimbabwe National Statistics Agency (ZIMSTAT) and ICF International.

Additional modeling work (for reference)

- Butler AR, Smith JA, Polis CB, Gregson S, Stanton D, Hallett TB. Modelling the global competing risks of a potential interaction between injectable hormonal contraception and HIV risk. *AIDS (London, England)*. 2013;27(1):105-113. doi:10.1097/QAD.0b013e32835a5a52.
- Jain A. Hormonal contraception and HIV acquisition risk: implications for individual users and public policies. Contraception, 2012;86(6), 645 – 652.
- Rodriguez MI, Gaffield ME, Han L, Caughey AB. Re-Evaluating the Possible Increased Risk of HIV Acquisition With Progestin-Only Injectables Versus Maternal Mortality and Life Expectancy in Africa: A Decision Analysis.Global Health: Science and Practice Dec 2017, 5 (4) 581-591; DOI: 10.9745/GHSP-D-17-00243

Funding

This work is made possible by the generous support of the American people through the U.S. Agency for International Development (USAID), provided to FHI 360 through Cooperative Agreement AID-OAA-A-15-00045. The contents are the responsibility of FHI 360 and do not necessarily reflect the views of USAID or the United States Government.

Collaborators and Thanks

Elena Lebetkin, MPH
Douglas Taylor, PhD
Xiaoming Gao, MS
Markus J Steiner, PhD
Kavita Nanda, MD, MHS
Laneta Dorflinger, PhD
Timothy Mastro, MD
Abdulmumin Saad, MD, PhD
Jennifer Mason, MPH
Lauren Maldonado, MPH

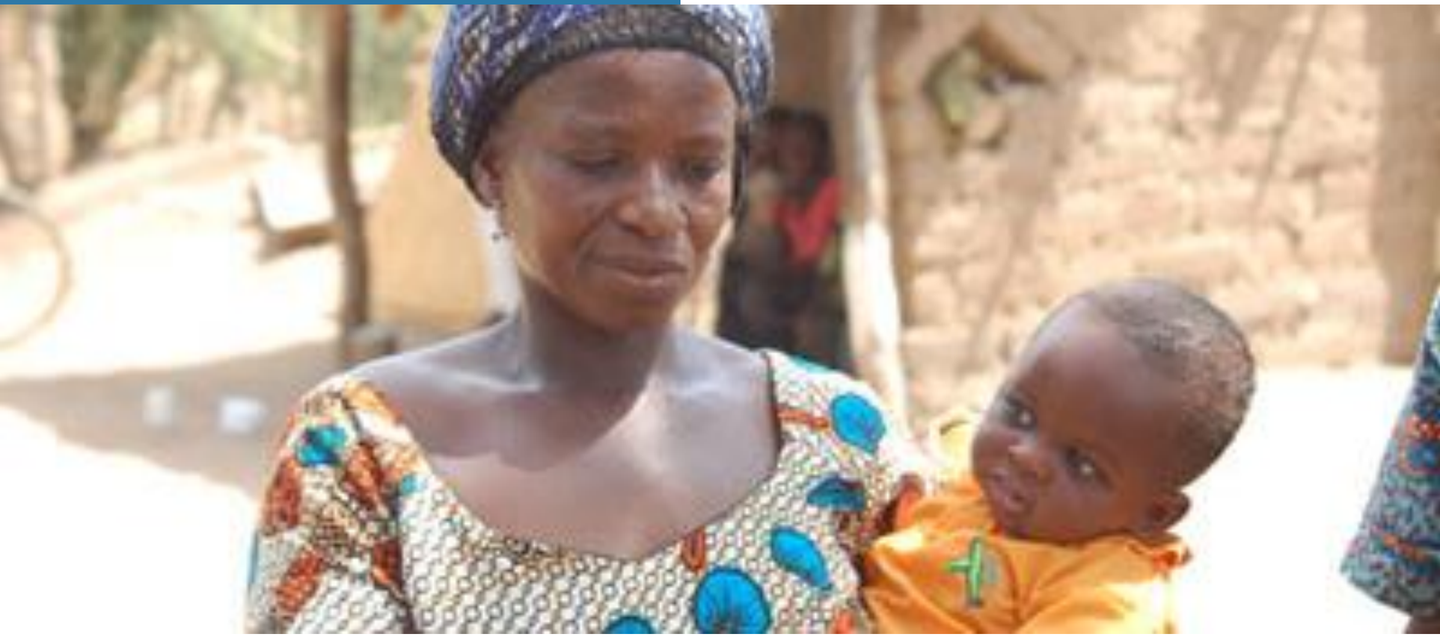

USAID

Envision FP

fhi360 THE SCIENCE OF IMPROVING LIVES
